# Predicting the Spatial Distribution of Wolf (*Canis lupus*) Breeding Areas in a Mountainous Region of Central Italy

**DOI:** 10.1371/journal.pone.0124698

**Published:** 2015-06-02

**Authors:** Elena Bassi, Stephen G. Willis, Daniela Passilongo, Luca Mattioli, Marco Apollonio

**Affiliations:** 1 Department of Science for Nature and Environmental Resources, University of Sassari, Sardinia, Italy; 2 School of Biological and Biomedical Sciences, University of Durham, Durham, United Kingdom; 3 Provincial Administration of Arezzo, Arezzo, Tuscany, Italy; University of Queensland, AUSTRALIA

## Abstract

Wolves (*Canis lupus*) in Italy represent a relict west European population. They are classified as vulnerable by IUCN, though have increased in number and expanded their range in recent decades. Here we use 17 years of monitoring data (from 1993 to 2010) collected in a mountainous region of central Italy (Arezzo, Tuscany) in an ecological niche-based model (MaxEnt) to characterize breeding sites (i.e. the areas where pups were raised) within home ranges, as detected from play-back responses. From a suite of variables related to topography, habitat and human disturbance we found that elevation and distance to protected areas were most important in explaining the locality of wolf responses. Rendezvous sites (family play-back response sites) typically occurred between 800 and 1200 m a.s.l., inside protected areas, and were usually located along mountain chains distant from human settlements and roads. In these areas human disturbance is low and the densities of ungulates are typically high. Over recent years, rendezvous sites have occurred closer to urban areas as the wolf population has continued to expand, despite the consequent human disturbance. This suggests that undisturbed landscapes may be reaching their carrying capacity for wolves. This, in turn, may lead to the potential for increased human-wolf interactions in future. Applying our model, both within and beyond the species’ current range, we identify sites both within the current range and also further afield, that the species could occupy in future. Our work underlines the importance of the present protected areas network in facilitating the recolonisation by wolves. Our projections of suitability of sites for future establishment as the population continues to expand could inform planning to minimize future wolf-human conflicts.

## Introduction

The wolf (*Canis lupus*) is an adaptable and generalist species. It is not especially habitat specific, can move over large areas, and can survive in many different environments, tolerating various degree of human disturbance [1–2]. Historically, wolves were widely distributed across the northern hemisphere but human persecution has greatly reduced and fragmented their range [3–4]. In Europe, after 1980, some remnant populations expanded into novel areas where human density was low and wild prey abundant [5–6]. Until relatively recently wolves across Europe were largely restricted to remote, scarcely populated, hilly or mountainous areas [1, 7–8], though with some exceptions in Spain [9].

The Italian wolf population represents one of the few surviving west European populations. Thus, it has great conservation importance at both a national and European level. The IUCN red list of threatened species classified the Italian peninsula population as vulnerable (category D1), and populations in the Alps as endangered (category D) (http://www.iucnredlist.org). Therefore, the restoration and recovery of this top predator is a conservation priority. In recent decades both the population size and range extent of wolves have increased in Italy. Wolves are recolonising their historical range, moving from the Apennines to the western part of the Italian Alps [10–11], and they are predicted to expand into the eastern Alps in the next ten years [12]. The Italian wolf population (easily identified by a unique MtDNA haplotype [13]) is a vital component of wolf restoration in Western Europe. Nevertheless, the small current populations remain susceptible to effects of demographic stochasticity [14].

As wolves are social carnivores and live in social units (packs), their density and territory configuration are a reflection not only of reproduction and mortality but also of group behaviour [1]. Moreover, social and physical factors influence individuals and their reproductive fitness, relative to the population in which they live and reproduce [15]. All these factors can affect the opportunity for new pairs to form and also their reproductive success [16]. Pup mortality is typically high during the first six months of life, and is related to the choice of home sites [17]. Home sites are defined as the combination of dens and rendezvous sites; rendezvous sites being the areas used by wolves to raise and leave pups after abandonment of dens. The locality of home sites can therefore be considered the focal point of a pack’s home range and the availability of such sites will affect the process of range expansion. Recently, several studies have related the choice of the home sites by wolves to variables such as climate, soil type, vegetation type, tree cover, human disturbance, and prey availability. However, most of these studies have been on North American wolf [18–22] populations with few comparable analyses from Europe [23–24].

Many modelling approaches are available to relate species presence-absence data to environmental variables [12, 25–28]. However, some species (particularly elusive species) can be overlooked during monitoring. Additionally, expanding populations are not at equilibrium with respect to potential explanatory variables rendering absence data problematic. Both of these situations are relevant to expanding wolf populations in Italy. In such a situation an approach that uses only recorded presences, such as maximum entropy modelling [29], provides an appropriate modelling framework [30,31].

Here we explore the importance of environmental and human-related variables in determining rendezvous sites in the Northern Apennines; an area of ~4 million hectares in the zone of recent wolf colonisation in Italy. We also model the suitability of the wider landscape of Italy for wolves, identifying suitable areas for wolves within the current range and potential areas for further expansion.

## Materials and Methods

### Study area

**Fig 1:**
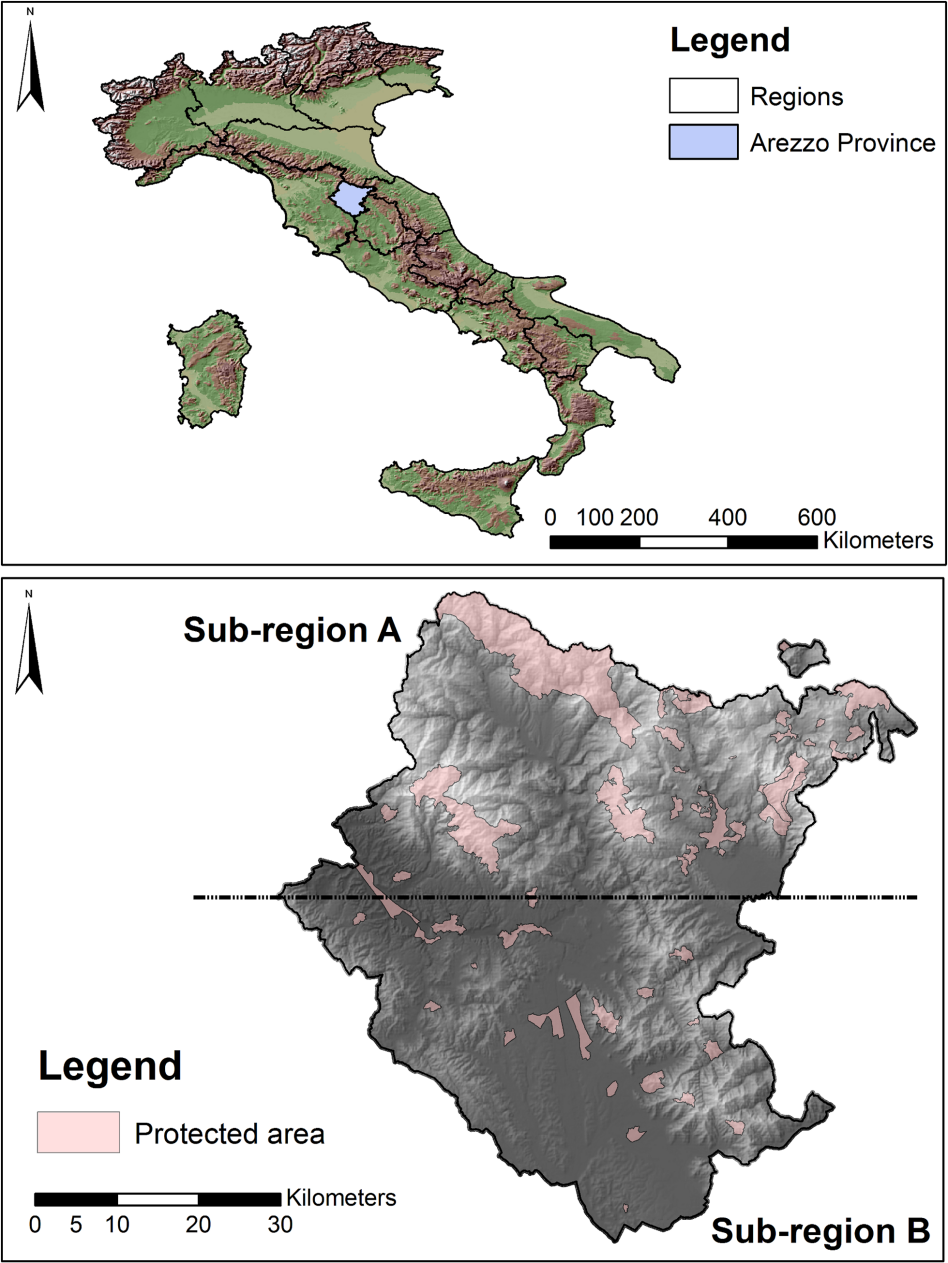
Study area. Map of Arezzo province, Tuscany, Italy. Pink shading indicates the protected areas. Grey shading represents elevation: lighter shades representing higher altitudes. The sub-region A was monitored from 1998 to 2010, sub-region B was occasionally monitored from 1998 to 2005, after which it was regularly monitored.

Arezzo province is an area of ~3,235 km^2^, located in Tuscany, Italy (Fig 1). Approximately 57% of the province is above 400 m a.s.l. with 7.4% being more than 1000 m a.s.l. The northern portion of Arezzo is mostly montane, including the Apennine chain and other secondary chains, with altitudes ranging from 300 to 1654 m a.s.l., and 66% of the area is forested. The southern portion comprises the lower course of the Arno River and Chiana Valley, the Chianti Hills and some low mountains; the altitude here ranges from 120 to 1081 m a.s.l., with only 32% of the area forested and approximately 50% comprising cultivated fields.

Forests in the province are predominantly deciduous with oaks (*Quercus cerris*, *Q*. *pubescens*) being the dominant species, along with beech (*Fagus sylvatica*) and sweet chestnut (*Castanea sativa*). Conifers comprise only a small component of forests (6.5%), represented principally by *Abies alba*, *Picea abies*, *Pseudotzuga menziei* and *Pinus* spp. The climate in the province is temperate-continental, with mean temperature ranging from 1.4°C in January to 24.9°C in July. The province supports a rich wild ungulate community including wild boar (*Sus scrofa*), roe deer (*Capreolus capreolus*), fallow deer (*Dama dama*), red deer (*Cervus elaphus*) and mouflon (*Ovis orientalis musimon*). Wild boar and roe deer are widely distributed throughout the study area, whereas the latter three species are more localised. Wild boar and roe deer represent the main prey species for wolves in the region [32–33], mainly occurring above 400 m a.s.l. Hunting of ungulates is forbidden in numerous localities within the study area (the mean size of no-hunting areas being 8.25 km^2^ and totalling 404 km^2^, Fig 1).

**Fig 2:**
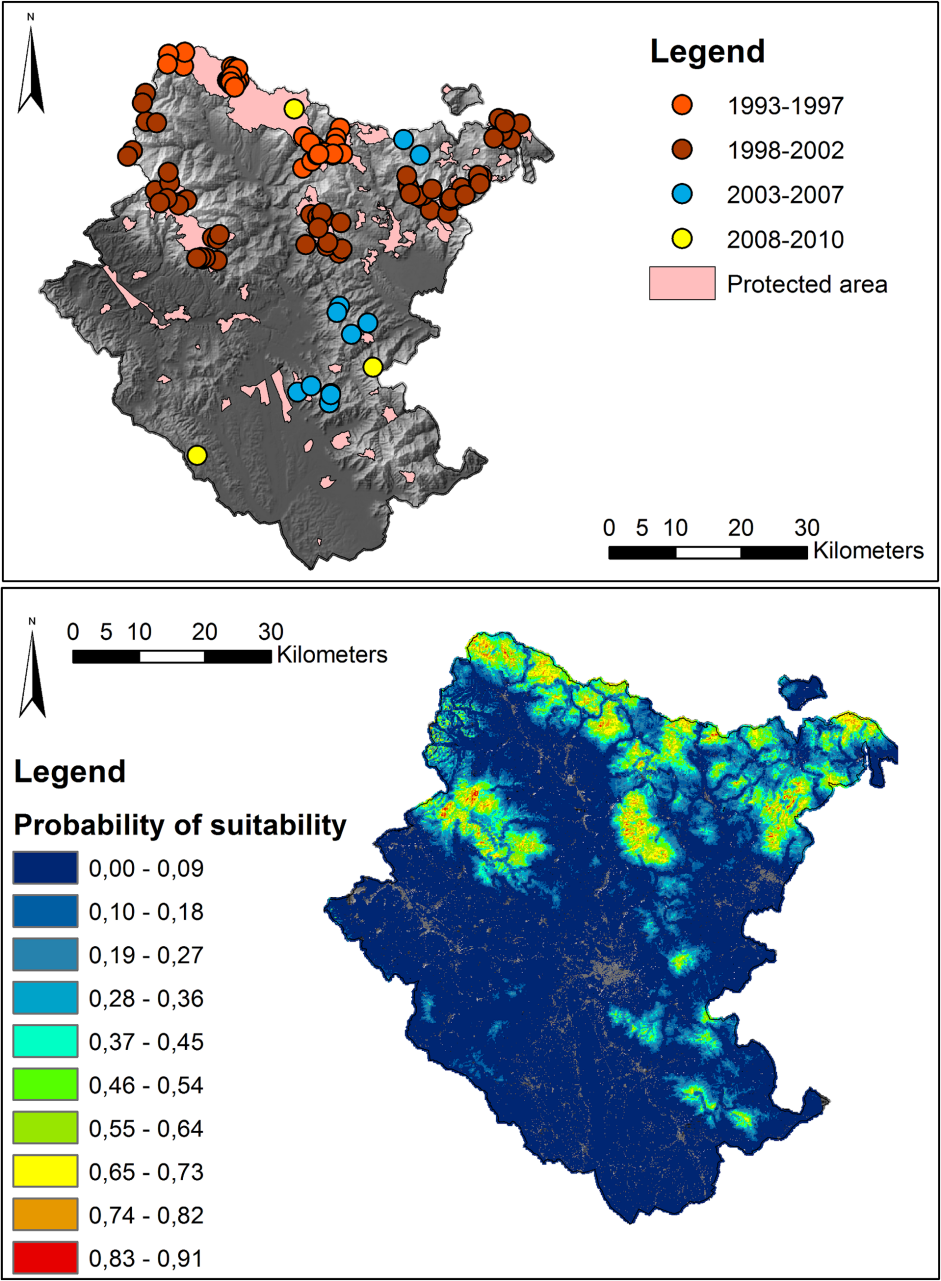
Rendezvous sites location and suitability map. Left: Locations of all rendezvous sites recorded during the study period (146 localities), in relation to protected areas. Red and orange dots represent the rendezvous that have been recorded in the period 1993–2003, while light blue and yellow dots represent the ones that have been recorded between 2003–2010. Right: Modelled mean suitability for rendezvous sites from the 10,000 model replications. Red indicates highest probability of occurrence, green intermediate probability and blues low probability.

The province is divided into 39 municipalities with a human population of circa 350,000 (107 people/ km^2^, ISTAT census of 2010). Urban settlements are restricted to lower altitudes and account for only 4.1% of the province. The road density (included paved roads, highways and forested roads) is 3.3 km/km^2^, with two-thirds of the roads concentrated in the southern portion. Cultivated areas are focussed around urban settlements and represent 42.3% of the province, comprising mostly plantations, pastures, and other cultivations (7%, 9.5%, and 25.8% by area respectively). Wolves remained in the highest mountain ranges of the northern portion of Arezzo throughout recent decades, with the first sign of wolves in the southern portion occurring in 2003 (Fig 2).

### Wolf monitoring

Wolves in Arezzo were monitored year round, integrating results obtained from snow-tracking, wolf-howling, molecular analyses of biological samples (scats, hairs, tissues, blood), and direct observation [34]. Permission to census wolves in the region was obtained through the Provincial Administration of Arezzo, and followed relevant national and international guidelines.

Rendezvous locations were monitored from 1993 onwards. Between 1993 and 1998, we collected data only in the Casentinesi Forests National Park (where wolf evidence was focused at that time). From 1998 to 2004, as wolves spread from the national park, we extended the monitoring to the northern portion of the province (sub-region A, Fig 1), monitoring the southern portion only occasionally, and then from 2005 monitoring the whole province (sub-regions A and B, Fig 1).

Rendezvous sites were located using howling playback response surveys, and following the saturation approach described by Harrington and Mech [35], and the protocol described by Gazzola et al. [36]. During a single night, two or more teams performed wolf howling surveys in adjacent areas of the province concurrently. We assumed that responding wolf packs were different if: (i) groups with pups were detected (see explanatory text below) by the same team in the same night in different valleys; (ii) groups were located by different teams in the same night in areas >5 km apart. The 5km threshold was chosen based on typical inter-pack distances in the province (unpublished data from 20 packs). In addition, if groups replied on different nights >5 km apart and had been identified as different packs (i.e. based on criteria (i) or (ii) above) during the previous year, then they were recorded as different packs again in the current year. The locations of rendezvous sites were calculated by triangulation from several points, adopting standard good practice in radio-tracking to minimise error [37]. Nonetheless, error remains in triangulated localities. To control for this, we modelled the characteristics of the rendez-vous site based on those of the 500x500m grid cell at which the triangulated point was central. The 500x500m cell adopted was appropriate given the error polygons association with tracking wolves using playback, i.e. when the detection is within a mile of the antenna and where playback occurs at high elevations to maximise detectability and precision (see [38]).

Every chorus and single response obtained during wolf howling surveys was recorded and the sonograms of registrations were analyzed using the software Raven Lite 1.0 [39]. By analyzing fundamental harmonics it was possible to count the minimum number of wolves that joined the chorus and, in the case of good-quality recordings, the presence of pups could be detected from their howl structure [40]. After September in their year of birth, pup calls cannot be reliably separated from adult calls. As we were interested in determining the environmental factors relating to rendezvous sites we included in our analyses only those chorus replies in which we could discriminate the presence of pups. Rendezvous site locations were pinpointed by triangulation and were overlaid onto 1:10000 scale digital maps (using ArcMap 9.3).

As a single pack can use the same rendezvous site for several years [24], to avoid overestimating the environment characteristics of a point recorded on numerous occasions (and hence minimising pseudoreplication), we excluded from our analyses repeat records from any sites (assuming that any records within 500 m constitute the same site).

### Enviromental and human-related predictor variables

We used seven classes of environmental variables, both categorical and continuous, as potential predictors of wolf habitat suitability. These variables were chosen based on their ecological relevance from other studies on den and habitat selection in wolves [8, 22, 24, 41–43]. The variables were: 1) habitat composition, divided into eight land-use categories (deciduous forest, coniferous forest, coppice forest, shrubbery, cultivated fields, urban settlements, paved roads, and unpaved roads); 2) distance from protected areas; 3) distance from water sources; 4) distance from roads (both paved and unpaved); 5) elevation; 6) aspect, divided into four temperature-related classes (the coldest, NE; the warmest, SW; and SE and NW) and 7) slope. The first four variables were extracted from a geographic information database of the Fish and Wildlife Office, Provincial Administration of Arezzo. Distance from protected area was measured from the edge of the nearest protected area; sites within a protected area having a distance of zero. The latter three variables were computed from a digital terrain model (available in the Tuscany Forest Inventory Map: http://web.rete.toscana.it/sgr/webgis/consulta/viewer.jsp). These data were represented as raster layers in a grid of 100x100 m resolution (1 ha) across an area of 6305 km^2^ covering the entire province.

### Habitat suitability modelling

To relate the occurrence of rendezvous sites (from the playback responses) to the landscape of the province we used the maximum entropy based machine learning program MaxEnt (version 3.3.3; http://www.cs.princeton.edu/~schapire/maxent, [29]). We used MaxEnt for our modelling framework for two principal reasons. Firstly, it is a presence-only model and does not require absence data, which are less reliably recorded for secretive and wide-ranging species such as wolves. Also, for expanding populations, as is the case here, absences may include suitable but uncolonised areas. Secondly, it is less sensitive than other approaches to the number of locations required to develop an accurate model [29, 44–45]; in some studies the added discriminative power of additional locations has been found to plateau at circa 50 records [44]. Although the use of ensemble projections based on multiple modelling approaches is often advocated when projecting distributions of species into independent situations [46], which is typified by the example of projections of species distributions under future climate change scenarios, our analyses are not projecting to independent situations. Instead, we aim to understand the drivers in one region, and to detect sites (within the region and within modelled parameter-space) of potential further expansion. To this end, we used MaxEnt as the sole modelling approach, as it has been shown to outperform other statistical approaches when tested on independent data [30]. Moreover, it has recently been shown that Maxent is largely equilavent to general linear models (GLMs) and point-process models, two potential alternative modelling approach [47].

For the model evaluation, MaxEnt produces both a threshold-dependent test, termed the “equalized predicted area” test, and a threshold-independent test, a ROC analysis [29]. The first test is based both on the omission rate, defined as the proportion of test localities that fall into cells predicted as unsuitable for the species, and on the “proportional predicted area”, defined as the proportion of cells that are predicted as suitable for the species [29]. The AUC (area under the curve) for a ROC (receiver operating characteristic) plot of sensitivity versus ‘1-specificity’ is used as a threshold-independent test of model performance; AUC being 0.5 when the model predictions were no better than random (for presence only data) and increasing to 1 for perfect discrimination.

Jackknife tests in MaxEnt were used to assess the relative contribution of individual variables to simulating the observed distribution and to identify the most informative variables in the final model. For the most informative variables we produced response curves to depict their relationship to modelled probability of occurrence of a rendezvous site.

We undertook cross-validation in MaxEnt, with 10,000 replications, which produced error estimates for ROC curves and average AUC values across models. We used default parameters for MaxEnt and we selected logistic output format, as generally recommended [48]. MaxEnt’s logistic output transforms the model from an exponential family model to a logistic model to avoid the possibility of probabilities of >1 [48]. For each variable in the model, regularized training gain represents the gain of the training data, regularized using the iterations performed by the model (n = 5,000,000), calculated either without the focal variable, or including only the variable of interest. The gain is a measure closely related to deviance. It starts at 0 and increases towards an asymptote during the run. At the end of the run, the gain indicates how closely the model is concentrated around the presence samples.

## Results

### Model evaluation and variable contributions

Over the sampling period we recorded a total of 146 rendezvous locations (Fig 2). Model performance, as indicated by the area under the receiver operating characteristic curve (AUC) value, ranged between 0.853 and 0.899 (mean value 0.876, SD 0.023), indicating that the environmental and human-related variables were very good descriptors in predicting rendezvous sites.

**Fig 3:**
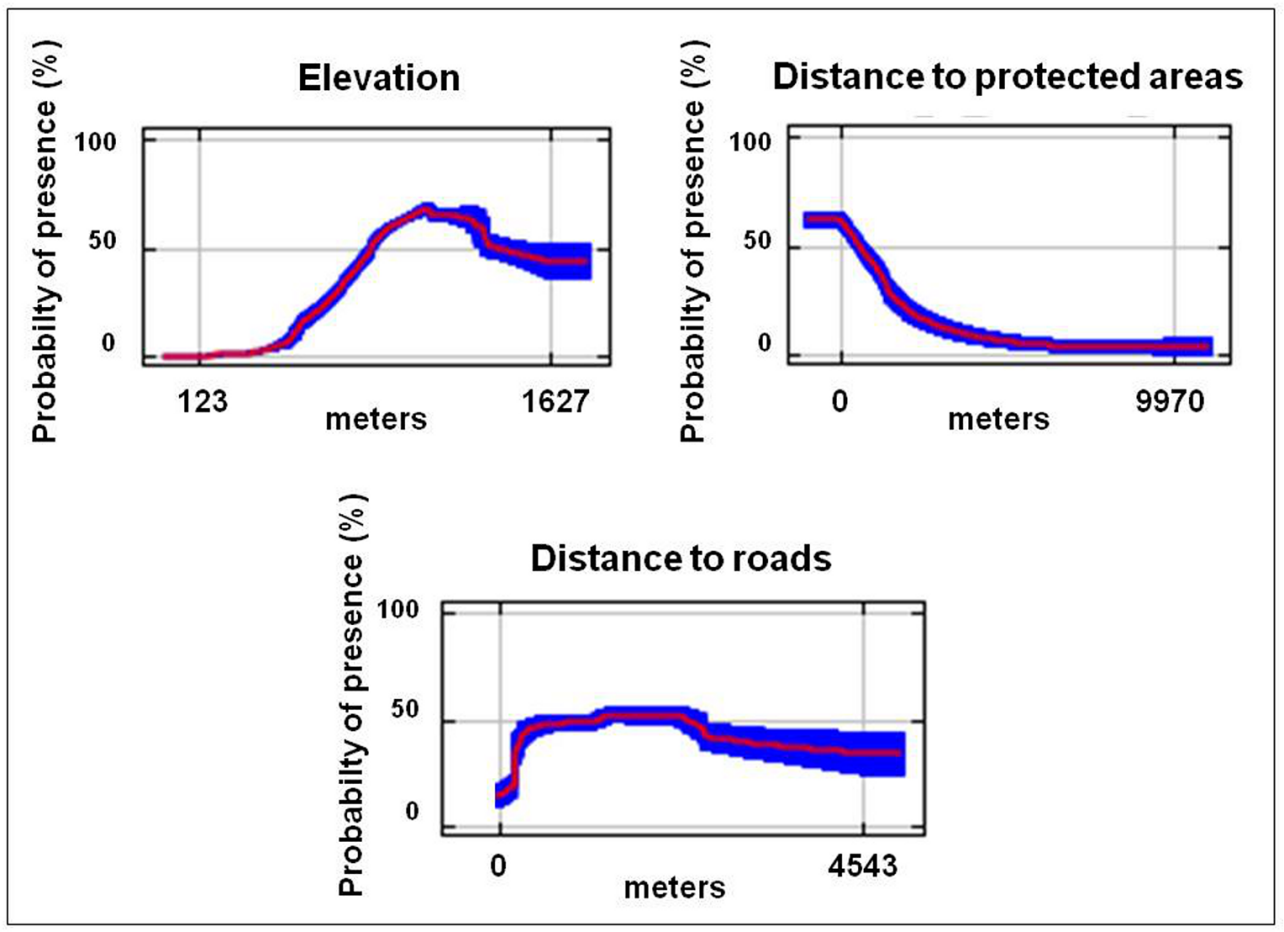
Response curves of the main variables. Response curves for the three most important environmental variables in the rendezvous site model. The curves show how the logistic prediction changes with each environmental variable, keeping all other variables at their mean sample value. Red lines represent the average trend for the variable considered, while the blue shading represents the standard deviation from 10,000 bootstrapped replications.

Elevation and the distance to protected areas were the two most important variables in the models; the former explaining 65.8%, and the latter 17.8% of explained variance (Table 1). Elevation and distance to protected areas were also the two variables that produced the highest gain when used in isolation and, when omitted, caused gain to decline most. Rendezvous sites were primarily associated with elevations between *circa* 800 m and 1200 m a.s.l. and within protected areas (Fig 3). Roads were negatively correlated with rendezvous sites, though distance to roads accounted for only 7.8% of explained variance (Fig 3).

**Table 1:** Summary variable importance and evaluation statistics for variables included in the MaxEnt model.

			Jackknife of regularized training gain	
Variable	Variable importance (%)	Permutation importance (%)	Without variable	With focal variable only
			(average log probability of the presence samples)	
Aspect	1.2	1.7	13.18	0.01
Distance to protected areas	17.8	26	11.44	0.46
Distance to rivers	1.5	3	12.97	0.02
Distance to roads	7.8	8.4	12.39	0.38
Elevation	65.8	55.7	0.99	0.96
Land use	4.6	3.4	13.08	0.33
Slope	1.4	1.9	13.09	0.31

### Simulated suitability for wolves

The most suitable areas (e.g. suitability values >0.6) for rendezvous sites from the models were located predominantly in the northern section of the province, mainly along the mountain chains, consistent with the known species distribution (Fig 2). In the southern section of the province the suitable areas were fewer in number and extent and tended to contain smaller areas of the highest suitability.

Comparing Fig 2A and 2B highlights that those areas of highest suitability were frequently within national parks or other protected areas. Regions occupied more recently, along the secondary mountain chains in the southern region, tended to have lower modelled suitability than the sites occupied up to 2002. It is also notable that unoccupied sites of highest suitability (>0.45) in the southern region overlapped considerably with protected areas, which occur predominantly at higher elevations. Interestingly, the most recent colonisations (since 2008, Fig 2B) have not occurred in areas of high modelled suitability.

## Discussion

### Model evaluation and variables’ contribution

The model of habitat suitability for rendezvous sites performed well in predicting recorded wolf distributions, with elevation and distance to protected areas being the two most important predictors of wolf breeding areas. Elevations associated with high probability of presence ranged between 800 m and 1200 m. These preferred altitudes may represent a combination of human avoidance [24] and protection against high summer temperature, particularly for the pups (minimum daily mean temperature and maximum daily mean temperature are 20–22°C and 25–32°C respectively, during the last 10 years in the July-September period, http://www.arsia.toscana.it). Moreover, this elevation range is characterized by a largely natural forested environment far from urban settlements, and has high densities of roe deer and wild boars (the most important prey species for wolves [33, 49–50]).

In this study, wolves chose to stay within, or very close to, protected areas during the pup raising period (Fig 3). Protected areas provide protection from direct persecution (as hunting is forbidden), human disturbance is low (there are a limited number of tourists and mushrooms or chestnut collectors in the summer period), and wolf prey resources are high. In the protected areas access is controlled and human activity is limited to daylight hours. The tendency to locate rendezvous sites and dens far away from humans and close to food resources is a well known aspect of wolf behaviour [24, 51–53]. Indeed, as suggested by Capitani et al. [24], the location of rendezvous inside, or at the border of, protected areas could represent a strategy for providing both reasonable protection to the pups and also high prey availability. This tendency of wolves to avoid areas with high human densities may be a strategy to increase survival, as the principal recorded causes of wolf mortality in Arezzo province are related to human activities (68% of recorded mortalities between 1990–2012, unpublished data).

Roads had little influence on the location of rendezvous sites. However, it should be noted that we performed an analysis combining paved and unpaved roads. Whereas the former may represent a substantial source of mortality due to traffic accidents (unpublished data), the latter are regularly used by wolves in their movements. Nonetheless, suitability for rendezvous sites did increase with increasing distance from roads (Fig 3), as noted in other studies [23–24]. In other studies wolves have been shown to avoid areas of high road density [8, 54–56]. Ghering [57] found that wolves preferred areas with a low density of roads, but that they frequently travelled close to trails and forest roads. Similar patterns have been observed in other large predators [58–59]. In a North American study, wolf dens tended to be located in roadless or in low road density areas, and were generally located more than 1 km away from paved roads [60]. The distance of rendezvous sites from roads in our study was similar, with a mean distance of 1140 metres (SD 820 metres) to the nearest road.

We found the proximity of rivers to be unimportant in the locality of rendezvous sites in our study, despite other studies finding that dens were often located close to water [3, 61]. This is probably due to water not being limiting in the study area, occurring widely and relatively homogeneously.

The wolf habitat suitability map presented here represents the first step in predicting the locality of rendezvous sites and, as a consequence, are informative in understanding habitat selection and the potential for future spread of the wolf across Italy. It remains to be seen whether the continued expansion of wolves in the region will occur predominantly in the areas we model as unoccupied, but suitable. It is noteworthy that two of the most recent expansion sites are in regions of low modelled suitability (yellow markers in Fig 2A), perhaps suggesting a shift in habitat selection more recently and highlighting one of the shortfalls of modelling habitat suitability using data from a species that is not currently at equilibrium with the environment. This raises the possibility that in future wolf-human conflicts may be more frequent than our models (that highlight avoidance of low elevations and areas of high human use) suggest. Suitable wolf habitat could be more widespread than our models suggest. Several studies have demonstrated that wolves can tolerate human presence and they can live close to humans [21, 62–65]. In these situations they tend to adopt a spatiotemporal segregation to avoid human presence and activities [53], as has also been shown to occur with brown bear (Ursus arctos) in Europe [58] and mountain lions (Puma concolor) in the USA [66].

It is notable that areas of moderate modelled habitat suitability are much more widespread than the highly suitable areas. Before the widespread loss of wolves across Italy (pre-1900), wolves were found in a much wider wide range of habitats, from sea level to the highest elevations [67]. That the northern portion of the province, especially the mountain chains, are simulated as more suitable than the southern lower elevations probably reflects the tendency of the relict Italian wolf populations to be restricted to, and hence recolonise from, mountainous, densely forested, and scarcely urbanized areas [67–68]. Wolf home ranges in southern and central Europe vary between 82–243 km^2^ [7], and have been estimated at 150 km^2^ in Dalmatia [69], and 197 km^2^ in Italy [62]. Combining this information with our areas of modelled suitability for wolves, we might expect at least 10 new packs to establish in the province if the population expansion continues.

To survive in human-dominated landscapes wolves need both protection from man and a healthy prey-base [70]. Maintaining healthy ungulate populations is necessary to minimise conflicts between wolves and other stakeholders, such as hunters and livestock owners. Hunters sometimes consider wolves as competitors for the same resource and tend to overestimate the predation of wolves on ungulates, despite many studies identifying hunting as the major source of ungulate mortality and the most limiting factor for ungulate populations [71–74]. In this context, maintaining healthy ungulate populations is important to reduce the perceived competition. Moreover, rich ungulate communities have also been shown to reduce depredation risk on livestock [71,75–76], but see also [77]. The tolerance of humans towards wolves depends mostly on their familiarity with their presence [63], and the reverse is also true of wolves towards humans [78–79].

The maps of simulated areas of suitability we have produced should permit a better understanding of potential sites of future spread and settling of wolves in this region. This, in turn, should facilitate management initiatives and education to reduce future potential human-wildlife conflicts and ensure the continued conservation of this important population of wolves [80–82].
